# From Neuroscience to Law: Bridging the Gap

**DOI:** 10.3389/fpsyg.2020.01862

**Published:** 2020-10-22

**Authors:** Tuomas K. Pernu, Nadine Elzein

**Affiliations:** ^1^Helsinki Collegium for Advanced Studies, University of Helsinki, Helsinki, Finland; ^2^Department of Philosophy, King’s College London, London, United Kingdom; ^3^University of Oxford, Lady Margaret Hall, Oxford, United Kingdom

**Keywords:** agency, causation, culpability, free will, liability, methodological dualism, neurolaw, prefrontal cortex

## Abstract

Since our moral and legal judgments are focused on our decisions and actions, one would expect information about the neural underpinnings of human decision-making and action-production to have a significant bearing on those judgments. However, despite the wealth of empirical data, and the public attention it has attracted in the past few decades, the results of neuroscientific research have had relatively little influence on legal practice. It is here argued that this is due, at least partly, to the discussion on the relationship of the neurosciences and law mixing up a number of separate issues that have different relevance on our moral and legal judgments. The approach here is hierarchical; more and less feasible ways in which neuroscientific data could inform such judgments are separated from each other. The neurosciences and other physical views on human behavior and decision-making do have the potential to have an impact on our legal reasoning. However, this happens in various different ways, and too often appeal to any neural data is assumed to be automatically relevant to shaping our moral and legal judgments. Our physicalist intuitions easily favor neural-level explanations to mental-level ones. But even if you were to subscribe to some reductionist variant of physicalism, it would not follow that all neural data should be automatically relevant to our moral and legal reasoning. However, the neurosciences can give us indirect evidence for reductive physicalism, which can then lead us to challenge the very idea of free will. Such a development can, ultimately, also have repercussions on law and legal practice.

## Introduction

According to a naturalistic, scientific, world view, reality is ultimately physical. Therefore, the human mind – our decision-making and behavior – must also be fundamentally physical. It would seem to follow, then, that the neurosciences, that study the physical basis of our minds, should be directly useful in understanding human decision-making and behavior, and should therefore also inform our moral and legal judgments.

Although this line of thinking is basically correct, it is all but clear how, exactly, neuroscientific evidence should bear on our moral and legal judgments. Here we outline a way of getting clearer on this by putting the question on the relevance of neuroscientific evidence to moral and legal reasoning in the more general context of metaphysics and the philosophy of science. Efforts to incorporate neuroscientific data into legal proceedings have had, at best, a mixed reception. We argue that much of the difficulty associated with the efforts to incorporate neuroscientific evidence in legal practice comes from a deeper problem of reconciling two radically different perspectives: ontological monism that pervades our scientifically based thinking about the metaphysics of mind, and methodological dualism that governs our folk psychological reasoning, and which cannot easily be eliminated within the practical constraints of legal contexts. While it is a mistake to suppose that neuroscientific data is wholly irrelevant to jurisprudence, or that it cannot in some cases help to determine legal responsibility, we need to exercise caution in attributing responsibility on the basis of such data. At worst, those drawing on such evidence in order to undermine claims of moral and legal responsibility might be accused of trading on unwarranted interactionist assumptions, where these involve a conflation of neural realizers of mental states with external causes of them. However, we argue that such cases of bad neuroscientific reasoning should not obscure the value of neuroscientific evidence in other cases. In particular, we need to make a distinction between changes in neural features that might plausibly be described as involving natural rewiring in the brain, and changes that we have adequate and independent grounds for classifying as involving external interferences to ordinary brain function. Here, we survey the way in which neuroscientific evidence has come to be increasingly utilized in legal contexts, evaluating the different ways in which such evidence is presented with the above distinction in mind. We highlight three different ways in which the neurosciences can, or cannot, be used to inform our moral and legal judgments. We think that the discussion on neuroscience and law has been conflating these issues, which explains why neuroscientific evidence has received a varied response in legal practice.

First, it seems that there is some quite obviously bad reasoning often done on the basis of neuroscientific evidence (see section on “Lessons From Physicalistic Monism and Methodological Dualism” below). It should be clear that just pointing to *some* neuroscientific data is not evidence of these neural correlates being the source of, or even relevant to, a given mental or behavioral phenomenon: we already know that brain-functioning is necessary for all mental and behavioral phenomena, and to assume otherwise would amount to committing a dualistic fallacy – the fallacy, in this case, of inferring the irrelevance of psychological notions on the sole basis of pointing to their neural correlates (cf. Pernu, 2011; Elzein, 2019). So, simply noting that there are some (homogeneous) neural correlates of the ways of behaving we deem immoral or illegal should not make one think that those correlates are causing that sort of behavior [cf. e.g., Glannon (2011) and Morse (2011a, 2015) in relation to discussion in section on “Lessons From Physicalistic Monism and Methodological Dualism” below].

Second, there are also better, and to at least some extent valid, ways of taking the neuroscientific evidence into account in our moral and legal reasoning (as discussed in section on “Basing Lack of Agential Control on Neuroscientific Data” below). This can, in principle at least, be done by first separating different mental faculties’ bearing on agential control from each other, and then showing that the functioning of some of the components essential for exercising those faculties has become dysfunctional for biological reasons. More precisely, in some cases we may be able to construct convincing evidence that there was some threat to agential control present due to neural factors on the basis that we have some independent evidence for a lack of control, and we can then point to a neural correlate for such a lack of control [e.g., Burns and Swerdlow (2003), Box 7 below]. Establishing such connections is practically very difficult, and we still have a lot to learn about the psychology and neuroscience of agential control, but there are no principled reasons why such connections could not be established.

Third, and contrary to some intuitions stemming from physicalist metaphysics, neuroscience cannot, by itself, disprove the ideas of agency and free will (as discussed in section on “Physicalism, Free Will, and Moral Responsibility” below). In cases where moral or legal judgments are based on neural evidence the conclusions follow precisely because we can compare cases of lack of control to normal control cases, and point to their neural differences (and maybe abnormalities). No such contrast can be made in more global worries concerning agency and free will, for we are not able to compare cases where free will is exercised to cases where it is not. There is, in other words, an often-neglected difference between establishing exculpating factors in a particular legal case, and appealing to neuroscientific data that would (if valid) undermine our notions of moral and legal responsibility more broadly. That is, we can use evidence that is meant to establish that no one is free to reform our legal practice as a whole, e.g., by casting a critical eye on the retributive functions of the criminal justice system, but such general arguments are not applicable to individual cases aiming to exonerate a particular defendant. Neurosciences can, and they constantly do, give us further indirect, inductive evidence for physicalism. And physicalism can, in turn, lead us to challenge the ideas of agency and mental causation, and consequently the very idea of free will. Such a development could, ultimately, also have repercussions on law and legal practice.

Let us make a few clarifications before moving on. The following discussion will focus solely on the impact of neuroscientific evidence on assessing the level of legal responsibility of a defendant in criminal law. More precisely, the focus here is on the issue of the *culpability assessment* of an individual legal agent (natural person) in criminal cases. Although this is the most typical context in which the connection of law and the neurosciences is discussed, it is important to keep in mind that the issue is in fact much broader, and the neurosciences can affect legal practice in various different ways, and raise a number of different ethical and legal concerns (cf. e.g., Greely, 2009; Farahany, 2016; Greely and Farahany, 2019). Neuroscientific evidence can also be used in civil cases (e.g., as a part of benefit claims), and neuroscientific methods can be used, not only in assessing the defendant’s mental state during the time of the criminal act, but also to improve our understanding of the behavior of other parties during court proceedings (i.e., witnesses, lawyers, judges, and juries), and to help us explain how the court arrives at its decisions (e.g., Schleim et al., 2011; Ginther et al., 2018). Neuroscientific evidence can also be used to inform our forward-looking judgments, e.g., in assigning punishment, in predicting and preventing criminal behavior, or in inducing neural changes (enhancement or impairment). Yet a different, but an important – and urgent – issue at the intersection of the neurosciences and law, is the question of how to regulate the use and data management of various different computer-brain interface devices, and the issue of the relevance of artificial intelligence to the practice of law in general.

The following will also rely on a very broad understanding of the notion of “the neurosciences,” encompassing e.g., anatomical, imaging (CT, EEG, fMRI, MEG, NIRS, PET, SPECT, X-ray), and behavioral considerations. “Neurosciences” will here also range across a variety of disciplines, from biology (phylogeny, ontogeny, physiology, genetics) to psychology, and the cognitive sciences in general. Although this does not depart from the general practice – as the discussion on the connection of law and the neurosciences typically relies on a very broad construal of “the neurosciences” – it is important to keep in mind that the field encompasses a wide range of methods and disciplines, and the distance between lower-level biological considerations and the higher-level psychological ones is significant. Indeed, the issue we are facing with respect to how to take neuroscientific considerations into account in our moral and legal reasoning can be seen to hinge on the very question of how our psyche should be understood to be related to its biological basis.

## Conceptual Preliminaries: From *Actus Reus* to *Mens Rea*

Intuitively, if one relies on a naturalistic view on the human mind, information about the neural basis of our decision-making and action-production should, in principle at least, have a bearing on our moral and legal reasoning. But why, exactly, would that be? What lies behind this intuition? Clearing up this conceptual landscape is the key to putting the empirical results and legal cases in their right places.

To zoom our focus, consider the following chain of conceptual dependencies:

legal responsibility → moral responsibility → free will → agency → causation

Here is a way of unpacking these connections. For you to be held legally responsible, a harmful event must have occurred, and that event must have resulted from actions that you wilfully and freely decided to perform. That is, the right sort of causal connection must hold between your decisions to perform certain actions and the outcomes of those actions, and “[b]ecause moral responsibility is tied to such a natural relation (i.e., causation), and because the law is tied to morality, the law also is tied to this natural relation” (Moore, 2009, p. 5). That causal responsibility is entailed by both moral responsibility (e.g., Glannon, 1997, 2002; Sartorio, 2007, 2016; Driver, 2008a,b, 2012; Braham and van Hees, 2012; Szigeti, 2014; Whittle, 2018; Willemsen, 2019) and legal responsibility (e.g., Hart and Honoré, 1959; Feinberg, 1962; Moore, 1984, 2009; Fletcher, 1998; Lehmann and Gangemi, 2007; Simester, 2017) is not only widely shared assumption of moral philosophy and legal theory, but also constitutes a fundamental element of our moral psychology (e.g., Shultz and Schleifer, 1983; Darley and Shultz, 1990; Sloman et al., 2009; Malle et al., 2014; Lagnado and Gerstenberg, 2017; Willemsen, 2019).

**FIGURE 1:**
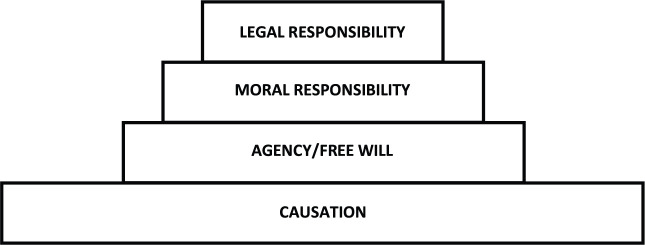
It is intuitive to think that legal responsibility is grounded in moral responsibility, which in turn requires agential responsibility, which is grounded in causal processes in the world. According to this simple hierarchical view, all the higher forms of responsibility are proper subsets of the lower level ones.

It might be intuitively appealing to construe the connection between these notions wholly hierarchically, in terms of proper subsets (Figure 1). That is, one could think that for there to be agency (ability to act) there must be causal processes in the world (only some of which are agential), and for there to be free will, there must be agency in the world (only some of which is free), and for there to be moral desert, there must be freely willed actions (only some of which we bestow with moral desert), and, finally, for there to be actions that call for legal consideration, these must be deemed as morally reprehensible actions (only some of which are serious enough to call for legal action).^1^

Although some such hierarchy must roughly hold, one can also point to gaps. Consider, most notably, the connection between legal and moral responsibility: we are sometimes deemed legally responsible for harmful events that we are not deemed morally responsible for – at least not without important qualifications. We may, more precisely, be held *financially responsible* for the harm caused by our action – we may be required to compensate for the damage that has been incurred – even if we would not be held *morally blameworthy* for the action; we may be found liable in tort law, even if no crime has been committed. So, although the two notions are clearly intimately connected, moral and legal responsibility do not form a straightforward hierarchy.

**FIGURE 2:**
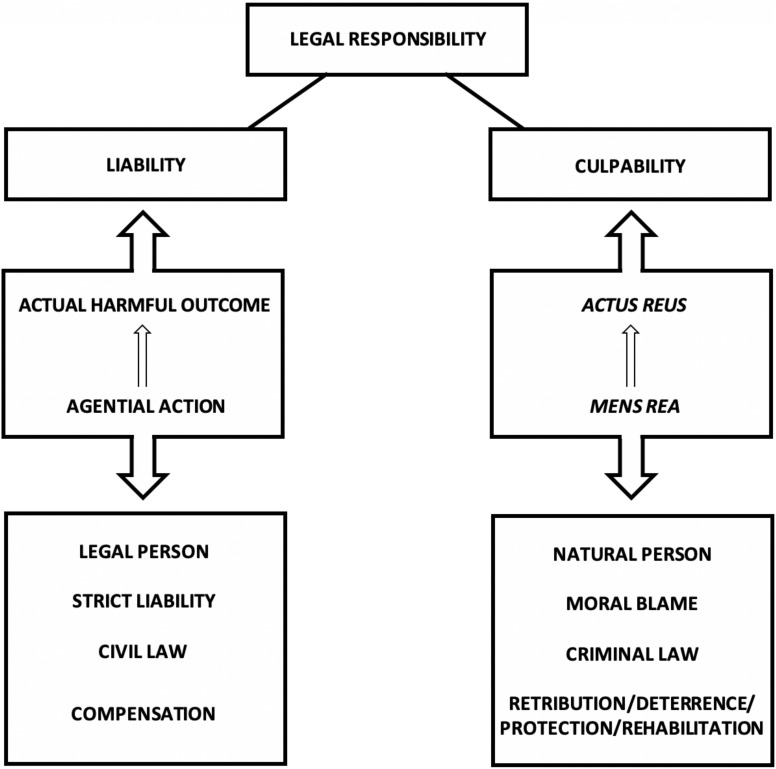
Legal responsibility can be interpreted to have two elements, or aspects: culpability and liability (Box 1).

What, then, separates these two types of responsibility from each other? Clearly: the agent’s state of mind. More precisely: moral blameworthiness requires, not only that there is a causal connection between the agent’s actions and the harmful outcome, but also that the agent strove purposefully (or at least negligently) to bring about the given outcome, and that she was aware of the harmful nature of the outcome. It is essential for moral blameworthiness, therefore, that a right sort of causal connection obtains between the agent’s mental states and the outcomes of her actions. Legal responsibility can, in turn, take place in the absence of such a connection. Thus, it is useful, it is here suggested, to separate *liability* from *culpability* (Figure 2 and Box 1).

**Box 1. The two aspects of legal responsibility.**

according to the distinction introduced here, legal responsibility can be attributed to an agent either on the basis of liability, or on the basis of culpability, or both (Figure 2). The necessary condition for an agent to be deemed liable (strictly liable) is a harmful outcome that has resulted from the actions of the agent; i.e., in order for an agent to be found liable, the actions of the agent must simply be causally connected to an outcome that is actually harmful (to another agent). In legal proceedings pertaining to liability, the status of the defendant is legal person (natural persons are a proper subset of legal persons). In case the defendant is found legally responsible in the sense of liability, she/it can be sentenced to compensate for the harm that resulted from her/its actions. For an agent to be deemed culpable (morally blameworthy), in contrast, no harmful outcome need have resulted from the actions of the agent; inchoate crime is also held as a crime. Thus, the necessary – and, *prima facie*, also sufficient – condition for culpability is the mere *actus reus* (the guilty act), which in turn presupposes *mens rea* (the guilty mind; criminal intent, encompassing criminal negligence) of the agent. In legal proceedings pertaining to culpability, the status of the defendant is natural person (legal persons cannot be deemed culpable). In case the defendant is found legally responsible in the sense of culpability, she can be sentenced in accordance with the penal code (which can be understood to function in terms of retribution and/or deterrence and/or protection and/or rehabilitation). In typical criminal cases both of these components of legal responsibility are present (for typically a harmful event has actually occurred), and guilty defendants are sentenced both to suffer penalties for their *acti rei* and to compensate for the harm that has resulted from them. However, these two components of legal responsibility are conceptually and historically distinct, and subject to different legal principles (which does not prevent them from seeping into each other in legal practice, e.g., in the notion of strict criminal liability, in assigning punitive damage compensation, or in the actual outcomes of *acti rei* having effect on sentencings in criminal proceedings).

Note that this distinction could in fact be stated even more starkly. One could hold that culpability (moral blameworthiness) actually has nothing to do with the actions of the agent and their outcomes – that it pertains solely to the agent’s mental states, namely her desires and intentions, and the decisions she makes on the basis of them – and that liability (legal blameworthiness *simpliciter*), in contrast, has nothing to with the agent’s mental states – that it pertains solely to the actions of the agent and the actual harm resulting from them (Figure 2 and Box 1). On this construal, moral and legal responsibility would be completely separate notions. In fact, this is how it used to be in early Anglo-Saxon law, for example, where legal actions were only carried out to determine the level and the subject of compensation of a harm that had been incurred, and the way that the harmful event occurred, and the intentions of the defendant whose actions resulted in the harmful outcome were simply irrelevant for the proceedings (Walker, 1968; Jacobs, 1971).

Today, however, the situation is quite the opposite: assessing the level of culpability of the defendant plays a major role in criminal cases – the more severe the case, the more so. Establishing the motive of a crime, for example, can be crucial for finding the defendant guilty for the crime. That is, for finding the defendant guilty of a crime – in the sense of finding her culpable for it – the defendant must be found to have acted on the basis of the right sort of reasons during the time of committing the crime. Moreover, and more strikingly, also inchoate crime is held, in severe cases, as a crime. That is, our current legal systems can, contrary to the old Anglo-Saxon one, focus solely on assessing the level of culpability of the defendant – even to the point of ignoring the issue of whether a harmful outcome actually resulted from the defendant’s actions. In fact, many claim that that is all that they should do, at least in criminal cases (e.g., Husak, 1987, 1998, 2007, 2011; Ashworth, 1993; Feinberg, 1995, 2003; Morse, 2004b; Alexander and Ferzan, 2009, 2018; Alexander, 2011; Enoch, 2014; Levy, 2015; Khoury, 2018).

The idea that we arrive at is the basic principle of criminal law: *actus reus non-facit reum nisi mens sit rea* – a harmful action without a guilty mind does not make one guilty. What this principle entails is that in order for a person to be found guilty of a crime, the right sort of causal connection must obtain (must be objectively shown to have obtained) between the defendant’s mental states (desires, intentions, decisions) and the harmful outcome of the defendant’s actions: *mens rea* – having a guilty mind – is necessary for culpability (Figure 2 and Box 1). Consequently, a critical element of criminal proceedings often pertains to the issue of establishing the *criminal intent* of the defendant: to be found guilty of a criminal act, the defendant must have made a conscious decision to act in a way that would bring about the harmful outcome in question – and the outcome must have resulted from this conscious decision.

Another way of putting this idea – the central idea of the criminal justice system – is that the focus of the proceedings is on the question of whether the outcome under scrutiny happened due to a given agent: whether the harmful event occurred or not was under the agent’s control. Central to the assessment of the level of culpability of an agent is, therefore, the notion of *sense of agency* – the issue of whether the occurrence of the harmful event was *up to the agent*. To be yet more precise, in order to be culpable, the mental states of the agent must have functioned as sources, or *difference-makers* for the outcome of the agent’s actions that have been deemed harmful. We can define “difference-making” in the following way: one event (the cause-event) is a difference-maker for another event (the effect-event) just in case the latter event would not have occurred had the former event failed to occur. For an agent to be culpable – for the right sort of causal connection to hold between the agent and the harmful outcome of her actions – the agent’s mental states (desires, intentions, decisions) must have stood in a difference-making relation to the outcome: the outcome would not have occurred, this idea requires, had the agent been in a different mental state (and would therefore not have performed the action that led to the harmful outcome). Let us suppose that having this sort of a relation between the agent and the outcome of her actions is a minimal requirement for finding the agent culpable for her actions.

Now, given this setting, it is quite easy to see how neuroscientific considerations might start to have a bearing on legal reasoning: they help us to assess whether the right sort of causal relation obtained between the agent’s mental states and the outcomes of her actions – they help us to assess whether the agent is culpable. Neural considerations might point to *neural dysfunctions* that could have disrupted the normal functioning of the neural basis of the relevant cognitive processes of the defendant. This could lead us to conclude that the required sense of agency did not take place – that whether the event deemed as harmful occurred or not was not up to the defendant – and the right sort of causal relation – the difference-making relation – between the agent’s mental states and the outcomes of her actions was severed.

Although it should be clear that the notion of sense of agency is crucial here, this notion lends itself to different interpretations. Let us call one view on it *subjective* or *internal*, and another *objective* or *external*. In some cases of loss of agency, we are speaking in former terms: that the person did not feel, from her own perspective, as if she had been in charge of the given events. In other cases of loss of agency, we have the latter view in mind: that the person was not, regardless of how she felt, in charge of the given events. Both of these types of considerations can play a role in assigning agency, and both views can be relevant to culpability assessments. One could, however, make a case for holding the latter to be more fundamental. Consider, for example, the fact that schizophrenia patients often report having control over things that they do not, in any objective or external sense, have control over (e.g., Voss et al., 2010). If you are under such a delusion, you are not (typically) considered to be culpable for the given harmful events. This would seem to suggest that external considerations can override reports about the subjective sense of agency, at least in assessing culpability: whether the occurrence of a harmful event was up to you is not, if you will, up to you.

The importance of separating these two different points of view on agency can be further demonstrated by considering how our moral and legal reasoning tackles intoxication. External considerations sometimes speak against the exemption of a defendant: even if the defendant’s sense of agency (e.g., memory, self-control) would have been significantly impaired when acting under the influence of alcohol or drugs, in typical cases that would not lead to us to relieve her of her moral and legal responsibility. Why? Because the agent had control over inducing those states on herself. The situation changes completely, of course, if the agent had become intoxicated and had acted precisely the same way, but she had gotten to that state by being drugged, without her knowledge, by somebody else. This suggests that our responsibility attribution practices track the ultimate agential source of our actions and states of consciousness whence the actions flow (Dimock, 2012).

**FIGURE 3:**
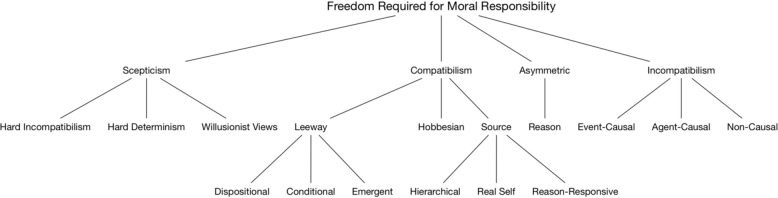
A taxonomy of the different philosophical accounts of free will [adapted from Elzein and Pernu (2017)].

This leads to another, perhaps the most fundamental conceptual distinction, which ought to be kept sharply in mind: we must separate the notion of agency *simpliciter* from the notion of free agency. That is, it is one thing to establish that a person has agency, and another, further thing, to establish free agency: as the hierarchy outlined above suggests, only some forms of agency can be marked as “free.” The notorious philosophical problem of free will pertains, first and foremost, to the latter notion: not many people are willing to strip us of agency – the ability to act – but many find it deeply problematic to attribute free agency – the ability to act freely – to us. What, then, is free agency? That is not an easy question, and no exhaustive answer to it will be given here. It should be noted, however, that both compatibilist and incompatibilist accounts, and various sorts of each, are out there (Box 2, Figure 3 and Table 1). We remain neutral to this dispute – the issue of whether determinism is compatible with free will – and merely point out that both accounts must give some story about how free, responsible agency differs from agency *simpliciter*. It should also be clear that the sort of external considerations pointed to above are crucial here: both accounts agree that external manipulation and coercion – the right sort of external forces – can rob us of our freedom and affect our assessments of culpability.

**Box 2. Different accounts of free will.**

**Skepticism: accounts holding that we lack free will**

**The Hard Incompatibilist View**

The sort of freedom required for moral responsibility is incompatible both with determinism and with indeterminism. So, however, the universe turns out to be, there can be no moral responsibility (Waller, 1990, 2011; Pereboom, 2001, 2014; Levy, 2008, 2011; Caruso, 2012, 2016, 2017, 2019; Shaw et al., 2019).

**The “Willusionist” View**

The sort of freedom required for moral responsibility is taken to be undermined by neuroscientific evidence, such as Libet experiments (Libet, 1985, Libet, 1994, Libet, 2002, Libet, 2003, Libet, 2004, Libet, 2006; Soon et al., 2008; Koenig-Robert and Pearson, 2019), which are taken to show that our conscious thoughts are not involved in producing our volitions (Wegner, 2002, 2004; Caruso, 2012).

**Compatibilism: accounts holding that free will is compatible with determinism**

**The Hobbesian View**

Freedom requires the ability to act on the basis of one’s choices, free from external constraints and impediments (Hobbes, 1651/1994). An external constraint is a factor that prevents one from carrying out one’s will; e.g., imprisonment might constrain an agent from acting as she wills. This view essentially rejects the notion of freedom of the will in favor of the notion of freedom of action; according to it free will is freedom to perform the actions we want to perform.

**Conditional Leeway View**

Popular view among a wide range of theorists especially in the first half of the 20th Century (Moore, 1903; Schlick, 1930; Ayer, 1954; Smart, 1961; Lewis, 1981; Berofsky, 2002). Freedom requires the ability to do otherwise, understood according to a conditional analysis of that ability. That is, an agent is able to act otherwise provided that the agent would have acted otherwise (or would be likely to have succeeded in acting otherwise) had she chosen to, or had she tried to.

**Dispositional View**

According to the dispositional analysis, freedom requires the ability to do otherwise, where this is analyzed in dispositional as well as conditional terms (e.g., Vihvelin, 2004, 2011, 2013). That is, an agent could have done otherwise if she would have done otherwise had she tried to, *and* if she could have tried to do otherwise. The ability to choose otherwise is then analyzed in dispositional terms: an agent could choose to do otherwise if she would choose otherwise were she placed in certain circumstances where the right sorts of triggers are present.

**Hierarchical Control View**

According to the hierarchical control view an agent’s first-order desires (e.g., “I want a cigarette”) must be distinguished from their second-order desires, desires regarding which first-order desires one has (e.g., “I want to not want a cigarette”). An agent’s *will* is defined as the first-order desire that actually moves one to action. An agent has a second-order *volition* when that agent has a desire regarding which of her first-order desires moves her to action (i.e., has a preference about which of her desires becomes her will). On this view, an agent has free will insofar as she is moved by the desires she wants to be moved by; acting in accordance with free will is essentially acting on the basis of desires that one endorses (Frankfurt, 1971).

**Real-Self View**

According to the real-self view, it is not enough that one is moved by second order desires. What matters is that one’s choices are in line with one’s most fundamental system of values – the “real-self.” These are the desires that one *rationally* identifies with (Watson, 1975).

**The Reason-Responsiveness View**

Fischer and Ravizza (1998) analyze moral responsibility in terms of “reasons responsiveness.” That is, the ability to respond to reasons in such a way that one would have done what there is most reason to do even if circumstances had been slightly different. The account parallels Nozick’s (1988) truth-tracking account of knowledge, according to which a belief counts as knowledge if it “tracks truth” in nearby possible worlds. Similarly, an agent counts as morally responsible (and hence having free will) if the agent’s decision-making mechanism tracks reasons.

**Emergent Freedom View**

An emergentist view on free will concedes, in line with incompatibilism, that indeterminism at the level of agency is necessary for free will and moral responsibility. However, the view also holds that indeterminism at the level of agency is consistent with determinism at the lower levels of reality. This is possible, according to the view, because the agency-level phenomena are multiply realizable at the lower levels, and the same agency-level phenomena could therefore have been realized by various different underlying physical bases (List, 2014, 2019).

**Asymmetric Accounts**

**The “Reason View”**

According to the “Reason View” moral responsibility requires the ability to do the right thing for the right reason (Wolf, 1980, 1990). This principle is asymmetric in its compatibility with determinism, with respect to moral desert (praise or blame). If an agent *has* done the right thing for the right reason, then, *a fortiori*, she is *able* to do the right thing for the right reason, so the condition is automatically met in the case of praiseworthy action. In contrast, if the agent has done something wrong, then she has failed to do the right thing for the right reason. In this case, the agent will only be responsible if she was *able* to do the right thing for the right reason. This is read as requiring the ability to do otherwise, holding the past the laws constant. Hence, praise is compatible with determinism, but blame is not.

**Incompatibilism: accounts holding that free will is incompatible with determinism**

**Event Causal Incompatibilism**

Event causal incompatibilists typically endorse similar conditions of free will to standard compatibilist accounts (such as the capacity to respond to reasons and to act in line with one’s deeper values). But they also require that one’s choices are not determined. On this view, it matters that one’s choices have the right sort of causal history (that they are sensitive to one’s values and reasons), but this history would not leave room for free choice unless one’s choice was also left open (where this is analyzed in non-conditional/non-dispositional terms). That is, free will requires the ability to do otherwise, as things actually stand, holding the past and the laws of nature constant (Nozick, 1981; Kane, 1996, 1999, 2011; Ekstrom, 2003; Franklin, 2011a,b, 2014, 2018; Lemos, 2018, 2020; see also Mele, 1995, 1996, 2006).

**Agent Causal Incompatibilism**

According to agent causal incompatibilism, freedom requires that the *agent* causes her own choices and actions, where this cannot be analyzed in event causal terms. On this view, the agent as a whole, rather than her mental states, must be one of the relata of causation, and she must figure as a direct cause of her choices and actions. The agent is a substance, an “unmoved mover,” able to influence her choices without being bound by any prior causal influence. The falsity of determinism is required, either because agent causation must involve non-conditional alternative possibilities (leeway incompatibilism), or because exercising free will requires one to be the “ultimate source” of one’s actions (source incompatibilism) (Reid, 1788/1969; Chisholm, 1964; Taylor, 1966; Clarke, 1993, 1996, 2000, 2019; O’Connor, 1995, 2002; Griffith, 2005, 2010; Steward, 2012).

**Non-causal Views**

According to non-causalists, free choices must be uncaused. That is, they must not be explicable in terms of the causal influence of prior events at all, and hence cannot be determined. Agent’s choices would not occur at random though, as non-causalists would require that choices must be *rationally* explicable. That is, an agent’s choice must made on the basis of reasons. It is denied, then, that reasons explanation is a species of causal explanation (*contra* Davidson, 1963). Reasons-explanations are taken to be *sui generis* (Ginet, 1990; McCann, 1998; Goetz, 2008).

**TABLE 1:** Commitments of different accounts of free will.

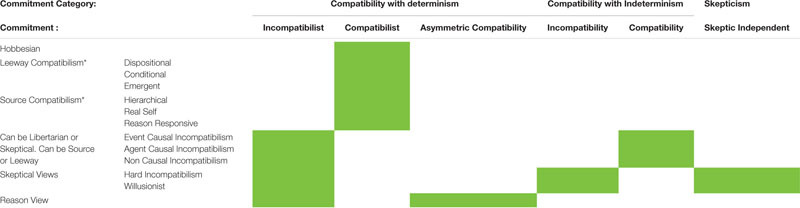

*Compatibilists are typically event causalists though other positions are possible.

To illustrate the importance of keeping these conceptual distinctions in mind in this context, consider the following example:

“To be found guilty in the U.S. legal system, a defendant must not only have performed a prohibited act, she must also have done so in a legally culpable state of mind. For example, if Mary suffers an unexpected seizure while standing on a subway platform and bumps into John, causing him to tumble to his death beneath the wheels of an oncoming train, Mary is not guilty of murder. Yet if she purposefully gave the same bump to John, intending his death by subway car, she would be. Neuroscience has sometimes been taken to suggest that the two scenarios are fundamentally the same and that therefore the legal outcomes should also be the same. Here is the reasoning: the motives that led Mary to push John purposefully onto the train tracks are products of her brain, which was in turn shaped by her genes and her environment, neither of which she chose. Accordingly, she is no more ‘responsible’ for her act when she intends it than she is when she has an uncontrollable seizure” (Jones et al., 2013, p. 17628).

One can now propose the following conceptual breakdown of this example. If Mary intentionally pushes John onto the train tracks, fully aware that that would result in great harm to John, most likely his death, and this is the actual outcome of her actions, then we should find Mary culpable for her actions and criminally liable for their outcome – we should find her guilty of murder. If, in contrast, Mary suffers a seizure, or if her behavior is determined by some other force outside of her control – if she herself had been pushed by someone else, for example – then we should deem her lacking *mens rea*, and not find her culpable for he actions and liable for the harmful outcome that actually occurred due to them. The question now is: to what extent should we let neuroscientific considerations affect our judgment in placing Mary into these two contrary slots? Should we think that the neurosciences reveal that she is – or that all of us would be in similar circumstances – pushed by her brain (together with her genes expressed in the given environment) to act in a certain way, and should she therefore be exempted from culpability, no matter what her internal states of mind had been? Naturally, we are prone to answer in the negative. But in seeing why the answer should, at least typically, be no, we need to get a clearer sense of when, and why, something counts as an external cause of an agent’s behavior and when we are merely giving an explanation of the way in which the behavior, and its psychological antecedents, are physically realized.

It seems clear that the key to unraveling all this is in pinning down the factors that lead us to strip a person of her agency. Being manipulated, or being physically pushed, by another agent will, in typical cases, make us conclude the person was not responsible for her actions and their outcomes. So, why, then, should biological factors sometimes be seen to play a similar role in stripping persons of their agency?

## Lessons From Physicalistic Monism and Methodological Dualism

The fundamental problem with connecting neuroscientific evidence to psychological and behavioral data, and drawing conclusions about causal relationships between the two, is the following: we know that all our mental states and processes, our personalities, desires, beliefs, and decisions to act this or that way, are grounded in our brains. Who we are, and what we do, is wholly dependent on our brains – without our brains, we, and our decisions and our actions, cease to exist. Therefore, just pointing to *some* neural correlates of our mental states or processes cannot, by itself, force us to conclude that those neural correlates, rather than the mental states that they ground, should be designated as the proper causes of our behavior.

This point is rather trivial: if we assume physicalism, the view that we are biological, and ultimately physical entities, as it has been assumed here, then we can be sure that we will always find neural correlates for our psyche and behavior. And yet, the neuroscientific literature is rife with studies demonstrating the structural and functional differences of the brains of various different types of people, such as string players non-string players (Elbert et al., 1995), Braille readers and sighted (Sterr et al., 1998), taxi drivers and non-taxi drivers (Maguire et al., 2000), musicians and non-musicians (Gaser and Schlaug, 2003), jugglers and non-jugglers (Draganski et al., 2004), pedophiles and non-pedophiles (Cantor et al., 2007), hetero- and homosexuals (Ponseti et al., 2007; Savic and Lindström, 2008), adolescence-limited and life-course persistent antisocial behavior (Carlisi et al., 2020), murderers and non-murderers (Sajous-Turner et al., 2019), among others. It is often unclear what the import of these studies is. We can assume, as we already knew that these people are behaviorally homogeneous, that their brains, that ground their psyche and behavior, are in some respects homogeneous. Therefore, such an observation does not, by itself, support the idea that the behavior of these types of people is somehow essentially – more than in other, “normal” people – dependent on such neural factors.

Consider, to connect this issue to the topic at hand, the example of the vivid social and behavioral gender differences in criminology: it is well-known that men commit substantially more crime than women, across cultures (e.g., Rowe et al., 1995; Burton et al., 1998; Carrabine et al., 2004; Walker and Maddan, 2013). We also know that there are numerous significant physiological differences between the two sexes, including neural differences. Should we now conclude that men are more prone to crime than women, and, more importantly, should we maintain that it is the brains of men, rather than their conscious decisions, that make them commit these crimes, and that men are therefore less culpable than women for their criminal behavior, or maybe exempt from it altogether? This is not a generally accepted way of reasoning. But why not?

The question, of course, is this: how do the observed correlations between certain types of neural and mental states arise? There are two different, but connected issues here. First, there is the metaphysical issue of how we should understand the mind and its neural basis to be connected to each other. Second, there is the more pragmatic, or methodological issue of how we should determine the right order of causes and effects in this context. The first issue is more fundamental. Suppose that dualism is the right metaphysical view. Suppose, in other words, that the mental and the physical are wholly distinct from each other. Then the issue of how the neuroscientific (physical) evidence should bear on psyche and behavior would not arise at all: the mental realm would evolve according to its own laws (if any). Or suppose, in contrast, that the mental and the physical are identical. In this case both neuroscientific and psychological evidence would be completely translatable to each other (as they are assumed to be referring to one and the same thing).

Both dualism and the identity theory seem unacceptable to scientifically informed common-sense: neither are the mind and the body wholly distinct, nor are our mental notions completely translatable to neural ones (and *vice versa*) (cf. Pernu, 2017). But what could the third way be? According to non-reductive physicalism – arguably the received view in current philosophy – the mental is dependent on the physical, but non-reductively so. That is, according to this view, there is always some physical (neural) basis for mental states, but the mental cannot be reduced to, or identified with, its physical basis. What, more concretely, could this then mean? Typically, the connection between the two is supposed to be understood in terms of *realization*: the mind – its mental functioning – is realized by neural processes. The often-used analogy is the distinction between software and hardware in computation (e.g., Block, 1995): the mind is, close to literally, a software run by the hardware of the brain; the mind is what the brain does. It follows from this that although the mind, to be able to function, must always be realized in some physical way – like a computational software must be run by some hardware in order to be functional – it can be realized in different physical ways – like a computational software can be run by different types of hardware. So, mental states must be physically realized, but they can be *multiply realized* by a variety of physical states, and are not therefore reducible to, or identical with them. This is how, according to this view, we can both preserve the intuition that the mental and the physical are distinct, but avoid the conclusion that dualism, at least of the classical substance kind, must hold.

This non-reductive way of conceiving the relationship of the mind and its neural basis is certainly very attractive. It is not without problems, however. Not only has the thesis of multiple realization itself been challenged (e.g., Bechtel and Mundale, 1999; Shapiro, 2000; Polger and Shapiro, 2016), but the view has also been argued to be unstable precisely due to its reliance on multiple realization: it has been argued that in order to account for the causal efficacy of mental states, they cannot be conceived to be distinct from physical states, must be assumed to be ultimately reducible to them – on pain of deeming mental states wholly epiphenomenal (e.g., Kim, 1989a,b, Kim, 1990, Kim, 1993, Kim, 1998, Kim, 1999, Kim, 2005; Papineau, 1993, Papineau, 2001, Papineau, 2009). Consequently, a vehement debate over the status of non-reductive physicalism rages on in current metaphysics and philosophy of science (Box 3). This is not the place to declare a verdict on it. Nor do we need to: whether it is the reductive or the non-reductive sort of physicalism that will ultimately prevail, they are both committed to the thesis that is now of interest – namely the idea that mental states and processes are always, in one way or another, neurally realized.

**Box 3. Non-reductive physicalism and the problem of causal exclusion.**

It seems natural for us to separate the mental from the physical, for various reasons (Pernu, 2017). For example, how you feel subjectively does not seem to be identical with the neural states that we observe to correlate with your feelings: although *you* might feel in a certain way – depressed, anxious, aggressive – it would not be correct to say that *your brain* has these feelings (even if these feelings would not be there without your brain). However, it also seems natural for us to hold that the mental and physical can interact – that your feelings and thoughts can have an effect on your body and on the course of events in the world surrounding you, and *vice versa*. But if we follow the first intuition, and set the two realms apart, it becomes difficult to see how they might interact.

How to resolve the tension between these two intuitions? Let us suppose – as both common sense and the current scientific consensus does – that neither the mental nor the physical can claim monopoly over reality. Let us suppose, in other words, that neither eliminative idealism nor eliminative materialism holds. If we assume that reality is neither purely mental nor purely physical, what options can we possibly have left? The currently popular view in philosophy suggests: *non-reductive physicalism*.

What, then, is non-reductive physicalism? Non-reductive physicalism holds that although the mental is dependent on the physical (in the sense that the former cannot exist without the latter), the former is neither identical with, nor reducible to the latter. But how can that be? How can something be dependent on something, but be neither identical with, nor reducible to it? Well, we can say that although no mental state can exist without being accompanied by a physical (neural) state, the reverse does not hold. That is, no particular physical (neural) state is necessary for a given mental state to exist. So, you cannot, according to this view, read off which particular neural state happens to hold from the psychological data alone, as a number of different neural states could function as physical bases of mental states. Although this view enjoys wide popularity in current philosophy of mind, it faces a well-known problem. According to the *causal exclusion argument* such a non-reductivist position is not stable, and will, when given a more detailed treatment, collapse into reductionistic physicalism (e.g., Kim, 1989a,b, Kim, 1990, Kim, 1993, Kim, 1998, Kim, 1999, Kim, 2005; Papineau, 1993, Papineau, 2001, Papineau, 2009). The source of this problem is in the basic assumption of all physicalism, namely in the assumption that the physical world is causally complete – that all physical effects have complete, sufficient physical causes. So, if every physical event in the world, that has a cause, has a physical cause that fully accounts for its occurrence, then postulating any mental causes appears wholly superfluous. It would thus seem inevitable that either mental states are epiphenomenal – that they are not doing any causal work in the world – or that they are identical with physical states – and as such states they would then be able to play the causal role we intuitively attribute to them.

The causal exclusion argument is currently under heavy debate. One popular non-reductionist strategy is to move the focus on the notion of causation at play in the argument, and criticize the idea that some events could be held “causally sufficient” for other events (e.g., Menzies, 2008, 2013, 2015; Woodward, 2008; List and Menzies, 2009; Raatikainen, 2010; Pernu, 2013b). If causation is understood in terms of counterfactual difference-making, rather than in terms of physical generation or production, the idea that mental states have autonomous causal power can be vindicated, according to this line of critique. However, there are a number of problems to address. There appears to be an equivocation on how the effect-events are individuated, for example, and the difference-making argumentation could be seen to lead to parallelism rather than interactionism (Pernu, 2013a, 2014a,b, 2016). And even more burningly, when the abstract philosophical argumentation is brought down to a concrete, neuroscientific level, the basic message of the causal exclusion argument appears to have bite again, and the mental and neural can be deemed identical, even if causation is understood in purely difference-making terms (Pernu, 2018).

The debate on how to relieve the tension between mental and physical causation, or higher and lower-level causation in general, continues.

Therefore, unless you are a card-carrying dualist, the simple fact that we can point to some neural correlates of our psyche and behavior should not come as a surprise. Yet, that is what often seems, implicitly or explicitly, to be the concrete conclusion of many brain imaging studies. Failing to keep clearly in mind the simple idea that mental states are always neurally based leads easily to the fallacious conclusion that it is discovered neural correlates that are causing the mental or behavioral differences that have been observed. But nothing of that sort can be established solely on the basis of the presented neuroscientific data. It is the biological function of the nervous system to be responsive to a variety of environmental cues, inanimate, animate, and social: it enables us to respond to the received stimuli in a flexible and appropriate manner. Different stimuli, together with a variety of preconditions at different levels of biological organization, shape our nervous system, which in turn forms the physical basis of our psyche and behavior. It is not so, therefore, that the changes in our psyche and behavior should be interpreted as being caused by neural changes, even if the two can be consistently linked.

To be more precise, in ignoring these basics, one easily falls prey to two different fallacies. First, there is the issue of the direction of causation. Once a neural correlate of a particular mental or behavioral feature is specified, one is easily led into thinking that the former is causally responsible for the latter – that the specified neural state *caused* the mental and behavioral changes that we observe. Typical neuroscientific data, imaging data in particular, is wholly statistical, and establishes only correlations between behavioral and neural variables, and the data alone does not therefore license a causal interpretation (cf. e.g., Tancredi and Brodie, 2007; Miller, 2010). Taking a more encompassing, metaphysical view on the issue does not give a shortcut to establishing causal conclusions. Although it follows from physicalism that mental and behavioral features are always neurally realized, and that the former are thus *constitutively dependent* on the latter, it would be wrong to think that the former must also be *causally dependent* on the latter – like, to use a very simple analogy, the bricks a house is made of are not a cause of the house. Note that even if one were to subscribe to the view that the mental reduces to the physical, or that the two are identical with each other, it would not follow that the former has to be causally dependent on the latter – quite the contrary: if the mental simply is the physical, then the two cannot be causally related, for identity is a symmetric relation (among other things) whereas causation is an asymmetric relation *par excellence*.

How, then, should we perceive the causal relationship between the two? That is not an easy question to answer. As already stressed, it might be altogether wrong to postulate any causal connection between the two (as the mental is realized, not caused by the physical). However, such a view would also go against the intuition that we often find it correct to say that the two are causally connected, e.g., when being knocked in the head causes you to become unconscious, or when being told that the house is on fire causes you to move your body out of the building. This is not the place to attempt to give a full account of how using such causal language – which is *prima facie* interactionist – can be made consistent with the monistic metaphysics of physicalism. It suffices here to make it clear that even in this physicalistic framework we need to give some such account: we need to explain why we sometimes point to physical (neural), and other times to psychological causes of our behavior. And more importantly: this precisely is the issue we are facing with neurolaw – the question of whether, in some cases, we should point to some biological *rather than* psychological sources of our behavior. That is, biological and psychological explanations of our behavior can, sometimes at least, be taken to be in a genuine pragmatic competition with each other.

This, it is here maintained, is at the heart of the problem of how to take neuroscientific considerations into account in our moral and legal reasoning. On the one hand, the discussion takes a certain kind of monistic metaphysics for granted, namely physicalism. On the other hand, we need to make sense of our talk of psychological vs. biological ways describing our behavior being mutually exclusive. Somehow, in other words, we need to accommodate our folk psychological dualism with metaphysical monism. Providing such an account is an ongoing philosophical project, and it is not the aim of this discussion to contribute to that. Here, we only point to this tension, and merely note that as long as we hold our folk psychological practices non-negotiable, and take our moral and legal reasoning to be resting on such practices, which seems plausible (cf. e.g., Lelling, 1993; Morse, 2003, Morse, 2004a, Morse, 2006, Morse, 2007, Morse, 2008, Morse, 2011a,2011b, Morse, 2013, Morse, 2015; Sifferd, 2006, Sifferd, 2018; Jakubiec and Janik, 2017; Hirstein et al., 2018; Moore, 2020), we must rely on *some*, albeit covert and unarticulated, criteria on how to demarcate between biological and psychological causal hypotheses. The monistic metaphysics of physicalism should therefore, in this context at least, be reconciled with methodological dualism.

It is, however, quite easy to point to one criterion that appears to play a crucial role in setting biological and psychological ways of explaining behavior apart from each other. This is precisely the issue of sense of agency: if a lack of sense of agency is detected, one is prone to shift from the psychological realm to the physical realm in designating the source of the given behavior. If, in other words, some events are not under agential control, then their causal sources should be traced back to somewhere other than to the psychological factors. And, as already stressed, here external considerations bear considerable weight. Compulsion, coercion, manipulation, and other such chains of events where the ultimate sources of the outcomes we happen to be interested in are designated to lie out of the reach of the agent, rob that agent of agency – at least the sort of free agency that is central to culpability assessments.

This brings us to the other fallacy that an uncritical treatment of neuroscientific evidence easily leads to. As external considerations bear a significant weight in demarcating between biological and psychological causal hypotheses in explaining behavior, one might be led into thinking that pointing to a biological (neural) abnormality would count as clear evidence for the presence of a factor outside the control of the agent. That is, one easily makes an inference from consistent neural differences to the claim that those neural features must be causing the observed behavioral differences. However, no such conclusion can be made solely on the basis of abnormality considerations. This is precisely because the basic function of the nervous system is to react and adapt to environmental cues; psychological and behavioral differences will always manifest themselves as some neural differences.

There is, however, a connection here that is worth highlighting. The notion of abnormality (consistent patterns of difference) is closely related to the notion of *dysfunction*. Pointing to dysfunctional neural features – to dysfunctional biology – would seem to give grounds for concluding that it is these neural features, rather than the mental features of the agent, that we should consider to be the proper causes of the agent’s behavior. Although this is no doubt the reason why abnormality considerations are prone to lead us to favor biological explanations over psychological ones, this observation will not take us much further in the analysis. The basic problem is that dysfunctional brains often reside in dysfunctional environments, and psyche and behavior can also be deemed dysfunctional. Again, therefore, pointing to a mere neural feature, a dysfunction in this case, cannot be made to justify the conclusion that this neural feature is the ultimate source of the behavioral features in question. We need independent reasons to hold the neural dysfunction to be caused by something outside the scope of the influence of the agent.

There is no doubt, however, that the notion of dysfunction is central here. Consider, in particular, the notions of disease and disorder, which are, according to a naturalistic reading at least, tied to the notion of dysfunction: a healthy organ or organism is one that functions properly, according to the way it’s supposed to function in light of its ecological role and evolutionary history (e.g., Boorse, 1975, 1976, 1977, 1997). Diseases and disorders are in turn something that we quite naturally hold to be autonomous with respect to the psyche and behavior of the agent: they are something that happen to an agent, and hence they are not something that the agent is in any way responsible for. This, of course, is the reason why considerations related to mental illnesses and disorders are highly relevant to culpability assessments.

So, we can presume that in pointing to neural abnormalities to demarcate between biological and psychological causal hypotheses in explaining behavior, the chain of reasoning goes from abnormalities to dysfunctions, and from dysfunction to illness or disorder, and then from illness and disorder to an entity outside the scope of the influence of the agent. Now, although this might be taken to be the correct description of the actual reasoning that lies behind the tendency to infer from neural data – the sort of data that points to a neural abnormality – to the conclusion that favors the respective neural features over psychological ones, doubts can still be cast on whether this sort of inference should be endorsed. The problem is that it is perfectly legitimate to question the apparent value neutrality of the notions of health and disease (cf. e.g., Sober, 1980; Kingma, 2007). In fact, the very notions of function and dysfunction are notoriously difficult to define in thoroughly naturalistic terms (e.g., Mayr, 1988; Allen and Bekoff, 1995; Garson, 2016). The core of the problem is that in deeming an entity (a property or a process) either functional or dysfunctional, we are always relying on pitting *right* and *wrong*, or *good* and *bad* ways of performing the function against each other; there is a gap between the way the function is actually performing, and the way it is *supposed to* – the way it *ought to* – be performing. But this sort of a gap – the gap between ought and is – cannot be closed, as the history of philosophy teaches us (Hume, 1738; Moore, 1903). If this indeed is the case – if there is no way of finding a neutral, naturalistic basis for correct and incorrect ways of functioning – then it is an illusion to assume that our moral and legal reasoning could be based simply on identifying neural dysfunctions.

This issue is connected to a pragmatic, methodological problem that we face in attempts utilize neuroscientific data in moral and legal judgments. In assessing the neuroscientific data, we are engaged with the project of connecting such data to psychological and behavioral data. Although we are easily led into thinking that the former is somehow the more primitive and fundamental of the two – precisely because we are relying, tacitly of course, on a monistic physicalist metaphysics – it is in fact on the basis of the psychological and behavioral data that we draw conclusions about the function of the neural features that are being studied. That is, it is not so that we deem some neural features dysfunctional on the basis of the neural data alone – such data will typically demonstrate only that these features are statistically abnormal. We deem the features dysfunctional on the basis of our prior understanding of the psychological and behavioral features with which the neural features are correlated. We first deem, for example, psychopathy or pedophilia to be psychological and behavioral dysfunctions, and we then proceed to identify the neural correlates of such behavioral patterns, after which we deem those neural features dysfunctional – not the other way around. Given that this is the general pattern in which neuropsychological reasoning, imaging studies in particular, proceeds, it is very problematic to start basing our moral and legal judgments on neuroscientific data. Not only are the psychological and behavioral considerations relevant, they are fundamental.

There is also an important trade-off to note: the more dysfunctional we consider the psychological and behavioral patterns to be, the less relevant the neuroscientific data related to these patterns is. If, for example, we are faced with psychologically and behaviorally clearly identified cases of mental illness or disorders (such as schizophrenia or psychosis), pointing to neural data correlated with these illnesses or disorders is bound to be thoroughly irrelevant to deeming the behavioral patterns in question as dysfunctional: we already know, based on the psychological and behavioral evidence, that the patterns are such. Consequently, in moral and legal reasoning – in making culpability assessments – related to such cases relying on the relevant psychological and behavioral evidence is wholly sufficient.

On the other hand, if we are facing less clear, or multifaceted psychological and behavioral patterns or personality traits (such as psychopathy or pedophilia), the neuroscientific data is bound to be irrelevant because that too is less clear and multifaceted. Morse (2011b, 2015) deems this the “clear cut” problem. To establish a reliable connection between behavioral and neural data, we need to rely on clearly defined behavioral variables. The less clear those variables are, the harder it will be to find robust and clearly defined neural correlates for them. But, when a sufficient clarity is achieved, and behavioral and neural data can be consistently connected, the neural data is bound to be irrelevant for our moral and legal reasoning with respect to the behavior – precisely because we already have a comprehensive and clear understanding of the behavior and deem it functional or dysfunctional wholly on its own merits.

Consider, finally, a concrete example of a case that exemplifies these problems we are faced with in trying to rely on neural evidence in our legal reasoning: the much-discussed case of *Roper v Simmons*, and the issue of whether we should hold the brains of adolescents underdeveloped, in relevant respects, and whether that should bear on our culpability assessments (Box 4). In this case, the defense tried to overturn a death-penalty sentencing ruling of a teenage defendant on the basis of arguing that adolescents have an impaired impulse control, due to their brains being underdeveloped, which should make us deem them less culpable than adults for criminal offenses.

**Box 4. Donald P. Roper, Superintendent, Potosi Correctional Center v Christopher Simmons.**

*Roper v Simmons* [543 U.S. 551 (2005)] was a landmark ruling in which the Supreme Court of the United States held that it is unconstitutional to impose a death penalty for crimes committed by adolescents under the age of eighteen. The ruling was made when the defendant, 17-year-old Mr. Christopher Simmons, had appealed his sentence to be executed, after a jury had found him guilty of the murder of Mrs. Shirley Crook. In the early morning hours of 9 September 1993, Simmons and his friend, 15-year-old Mr. Charles Benjamin, broke into Mrs. Crook’s home, in Jefferson County, Missouri, as a part of a plan to commit burglary and murder. After Crook awoke upon hearing the pair and called out, Simmons and Benjamin entered her bedroom, tied her hands up, and covered her mouth and eyes with a duct tape. They then drove the victim to the Castlewood State Park, and pushed her off a railway bridge into the Meramec River, causing her death by drowning. They stole the victim’s purse, which they later threw into the woods. The proceeds of the crime were reported to have added up to $6. Both defendants were convicted for the crimes. Benjamin was sentenced to life in prison, but Simmons was given the death penalty. Simmons filed a series of appeals in the years that followed, and the case worked its way up both state and federal courts, with all of them upholding the death penalty. Eventually, in 2002, the Missouri Supreme Court stayed the execution while the U.S. Supreme Court decided *Atkins v Virginia* [536 U.S. 304 (2002)], which dealt with the issue of the death penalty for the intellectually disabled. As the U.S. Supreme Court did in fact rule that executing the intellectually disabled amounted to a cruel and unusual punishment, violating the 8th and 14th Amendments of the U.S. Constitution, the Missouri Supreme Court decided to reconsider Simmons’ case, subsequently leading them to rule, 6-to-3, that executing minors would also amount to a cruel and unusual punishment. However, an earlier ruling of the U.S. Supreme Court, in *Stanford v Kentucky* [492 U.S. 361 (1989)], had decided that executing minors was not unconstitutional. This prompted the lawyers for Missouri, and Mr. Donald P. Roper, the superintendent of Simmons’ correctional facility, to argue that the Missouri Supreme Court was contradicting the U.S. Supreme Court. Therefore, in *Roper v Simmons* the question was, in effect, whether adolescent defendants should be considered analogous in relevant respects to the intellectually disabled in capital crime cases. Evidence was presented to the court aiming to establish that human brains, the prefrontal areas in particular, continue developing until the early twenties, and that minors are, for these precise neurodevelopmental reasons, biologically impaired in their capacity for moral reasoning and self-control. It was then argued that executing minors amounted to a cruel and unusual punishment, violating the constitution. The U.S. Supreme Court did in fact overrule, 5-to-4, their earlier *Stanford v Kentucky* decision, and concluded that it is unconstitutional to impose a death penalty for crimes committed by minors, resulting in overturning death penalty statutes in 25 states. While neuroscientific evidence of the relative underdevelopment of the brains of adolescents was presented to the court, and while the arguments drawing on such evidence did receive significant attention, both from the experts and the public, the final verdict actually gave significantly more weight to social, psychological, and common-sense evidence, with the dissenting justices expressing skepticism of the relevance of neuroscientific evidence to legal procedure. However, the case demonstrated the potential impact of neuroscientific evidence to legal proceedings, and it was central in setting off the current discussion on the role of neuroscientific evidence in jurisprudence.

**FIGURE 4:**
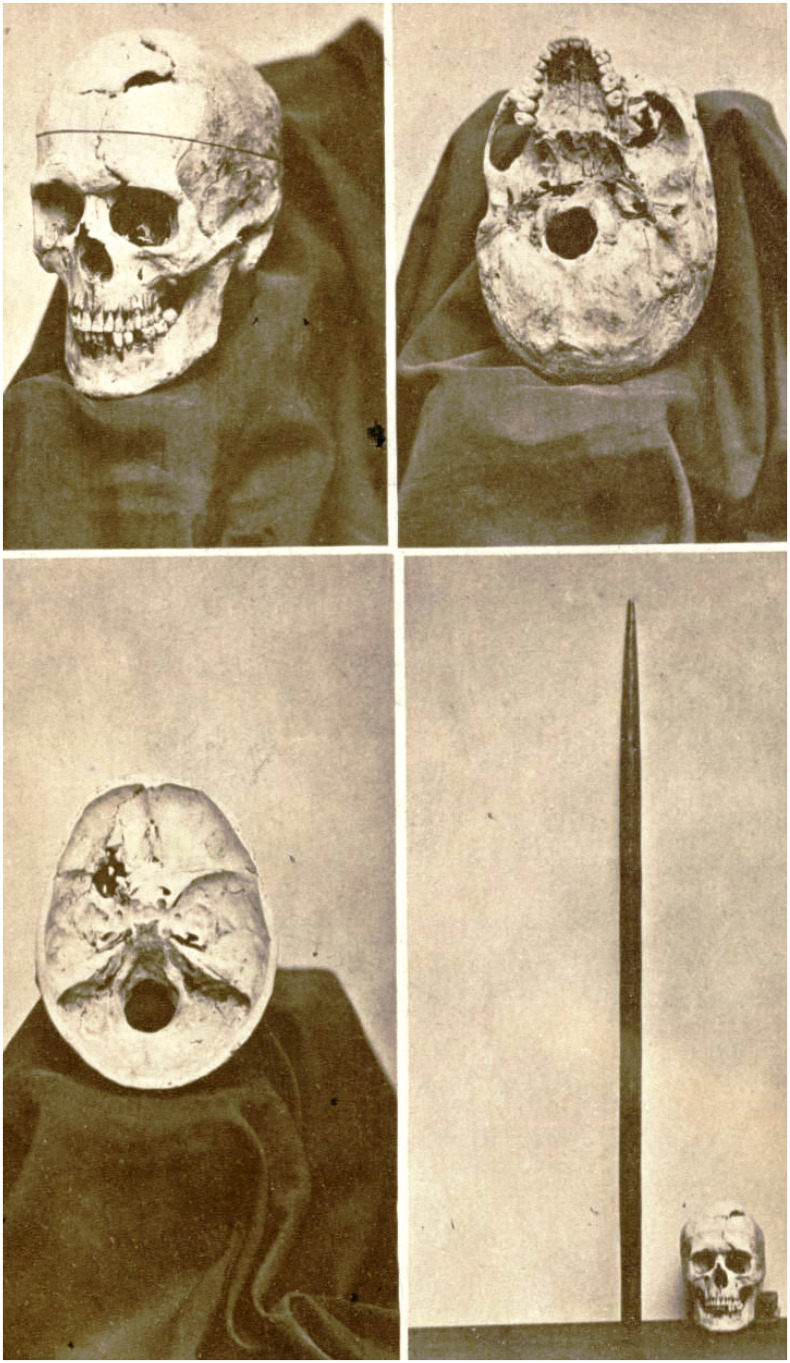
The skull of Phineas Gage and the iron bar that pierced his skull in the accident on 13 September 1848, as shown in a catalogue of the Warren Anatomical Museum, Harvard Medical School (Jackson, 1870).

The discussion on neurolaw often takes a critical view on the reasoning presented in the case (e.g., Glannon, 2011; Morse, 2011a, 2015). We can now see why that is: it exemplifies the very problems that were just reviewed in connecting neural data to psychological and behavioral data. The basic problem is, in other words, that we already knew that adolescents are different to adults in a number of ways, but in terms of impulse control, sensitivity to peer pressure, and sense of responsibility in particular. That is, we knew this based on our psychological, social and cultural understanding of adolescents, and as we knew that the psyche and behavior of us all, adolescents and adults alike, is dependent on our brains, it should not come as news to us that we can point to some neural differences that function as a physical basis of the psychological and behavioral differences that we observe. It seems that in *Roper v Simmons* the defense tried to make a case for causal explanation of the actions deemed harmful in biological, rather than in psychological terms, by appealing to neuroscientific evidence, in order to make the court infer a diminished sense of agency from this, and then make an inference to diminished culpability of the defendant on the basis of this. However, such a chain of reasoning is not valid, precisely because simply pointing to neural differences between adolescents and adults should not make us conclude that a given action has biological, rather than psychological, causal etiology. Some independent reasons should be given to think that the biological features referred to are dysfunctional, and that this is due to biological rather than psychological factors. But as such reasons were not presented, all we are left with is the affirmation of the triviality that adolescents and adults have psychological and behavioral differences that are correlated with neural differences. Consequently, although the court did put some weight on the neuroscientific evidence presented by the defense (Carbone, 2011), its final decision was largely independent of it.

## Basing Lack of Agential Control on Neuroscientific Data

The fundamental problem of utilizing neuroscientific evidence in our moral and legal reasoning stems from the fact that all decision-making and action-production is neurally based. It seems to follow from this that all our actions, including the *acti rei* that we find morally and legally concerning, are neurally caused. So, if simply pointing to such neural factors were to constitute a valid basis for exoneration, *all* our actions would become exonerable: “since all behavior is caused by our brains, wouldn’t this mean all behavior could potentially be excused?” (Rosen, 2007). This is not how our actual moral and legal reasoning works. Typically, we are judged to be morally and legally responsible for our actions. But sometimes, in some cases, pointing to neural factors does have an effect on our moral and legal reasoning.

So, how to demarcate between good and bad ways of taking neuroscientific evidence into account in our moral and legal reasoning? Let us approach this question by considering some actual, concrete cases where it would seem natural for us to point to some neural factors as sources of behavioral patterns that we find morally and legally concerning (Boxes 5–7).

**Box 5. The case of Mr. Phineas P. Gage.**

Perhaps the most famous historical case demonstrating a dramatic change in personality and agential control is the case of Mr. Phineas Gage [1823 (presumed) – 1860], a 25-year-old railroad worker, who, in 13 September 1848, endured a devastating accident when an iron rod blasted through his head (Harlow, 1848, 1868; Macmillan, 2008). The rod entered through the left side of Gage’s face, breaking his upper jaw, pushing directly through his forehead (destroying his left ventromedial frontal cortex), and protruding out through the top of his skull (Figure 4). Astoundingly, Gage survived the incident. However, the physicians treating him chronicled dramatic personality changes, including a lack of restraint, and a marked decrease in his ability to control his impulses. While this case has become legendary in psychology literature, it has also been apparently subject to notable embellishments. Nonetheless, the case still seems to provide a clear example of changes to behavior and capacity for self-control that likely result from brain injury (Damasio et al., 1994; Macmillan, 2000, 2008). Phineas Gage’s skull is now on display at the Warren Anatomical Museum, Harvard Medical School (Figure 4).

**Box 6. The case of Mr. Charles Whitman.**

Mr. Charles Whitman (1941–1966) was a student at The University of Texas, with a previous career in the Marine Corps. He was largely described as a popular and intelligent young man by those close to him. On the night of 31 July 1966, Whitman drove to his mother’s house and stabbed her to death. He then went back home, and stabbed his own wife to death. That night Whitman typed notes in which he proclaimed to love his mother and his wife very much, despite brutally killing them both. He also expressed his inability to understand or explain his own behavior, and requested that an autopsy be performed in order to determine whether there was some biological cause for his actions, which might also explain the constant headaches he had been suffering. Next morning, 1 August 1966, Whitman, a skilled marksman, climbed to the 28th floor of the tower of the main building at The University of Texas at Austin, and began shooting indiscriminately. He ended up killing fourteen people and injuring a further 31, before being killed by a police officer. In an autopsy conducted after his death it was discovered that he had had a brain tumor. This was classed as a glioblastoma multiforme tumor the size of a pecan, located beneath the thalamus, but potentially impacting the hypothalamus, the temporal lobe, and the amygdaloid nucleus. Many have dismissed the tumor as being unlikely to explain his behavior, in line with the original conclusion of Dr. Coleman de Chenar, who first performed the autopsy. Nonetheless, Texas Governor John Connally’s committee, comprising thirty-two experts, argued that the case is inconclusive (Texas Governor’s Committee and Consultants, 1966). Those doubtful about the significance of the tumor point to a number of psychosocial factors, such as Whitman’s troubled relationship with his father, his anger at his life situation, feelings of personal failure, and his domineering behavior toward his wife (Lavergne, 1997). But such explanations do not obviously help to account for his explicit claims not to understand his own behavior, his explicit record of struggling to control impulses he failed to recognize as his own (as chronicled in his diaries and suicide note), and the fact that he was actively trying to seek psychiatric help for his condition. There is also a great deal of neural evidence that does in fact link disruptions to the amygdala and temporal lobe to aggressive behavior, rage, and poor impulse control (e.g., Damasio, 1994; Grafman et al., 1996; Anderson et al., 1999; Bechara et al., 2000; van Elst et al., 2000; Mobbs et al., 2007; Schneider and Koenings, 2017).

**Box 7. The case of recurring brain tumor and pedophilia.**

Burns and Swerdlow (2003) describe the case of a 40-year-old male who began to develop a strong interest in pornography, including child and adolescent pornography, and began to make sexual advances toward his prepubescent stepdaughter (leading to a conviction for child molestation). The man had no previous record of sexual interest in children. His behavior was coupled with a broader inability to control his sexual impulses, and with attempts to solicit sexual contact in inappropriate circumstances. The person was eventually admitted to hospital, on the basis of complaining about a headache. While at the hospital, he reported balance problems, displayed marked difficulties with some of his movements, and appeared unconcerned that he had urinated on himself. He also had suicidal thoughts, reported fearing that he would rape his landlady, and attempted to solicit sexual favors from female nursing team members. Magnetic resonance imaging (MRI) scans revealed a tumor displacing his right orbitofrontal cortex and distorting the dorsolateral prefrontal cortex. Upon removal of the tumor, his bodily control returned to normal, and after participating successfully in a Sexaholics Anonymous program, he was believed to pose no more threat to his stepdaughter, and was able to return home. A year later, he again developed a consistent headache and began secretly collecting pornography. MRI imaging showed tumor regrowth, and once again his symptoms abated after its removal.

Consider, first, the historically important, and much discussed case of Mr. Phineas Gage, whose personality changed dramatically after a serious brain injury due to an accident in 1848 (Box 5). According to the sources of the time (Harlow, 1848, 1868), the once a hard-working, responsible, and much-liked man became, after the accident, explicitly anti-social and could not return to his previous job. His personality changed completely; “Gage was not,” his friends would say, “Gage anymore.”

Consider, next, the case of Mr. Charles Whitman, who indiscriminately shot at victims on a campus of The University of Texas at Austin in August 1966 (Box 6). Before the killings, he documented having “irrational thoughts,” and feeling that he does not “understand himself.” He requested that an autopsy be performed after his death to determine the cause of his thoughts and feelings, and his uncontrollable urge for aggressive behavior. A brain tumor was in fact later found, and it is plausible to suppose that Whitman may have suffered diminished control due to the tumor.

Consider, finally, the case of a man described by Burns and Swerdlow (2003), who developed uncontrollable and uncharacteristic sexual urges, that included pedophilic tendencies (Box 7). This led into him being arrested and convicted. Later, a brain tumor was found, and removed, which resulted in the disappearance of his criminal behavior.

One important thing to note is that anatomically all these cases involve some neural changes located in the prefrontal cortex (PFC). The function of PFC, in turn, has been associated emotional regulation and social behavior. Increase in aggressive behavior has been linked to PFC damage in Vietnam War veterans (Grafman et al., 1996), and reduction in PFC brain volume has been reported in patients diagnosed with anti-social personality disorder (Raine et al., 2000), aggression disorder (Woermann et al., 2000), and pathological liars (Yang et al., 2005). Imaging studies have revealed abnormalities in PFC function in violent people (Volkow and Tancredi, 1987; Chester et al., 2017) and convicted criminals (Raine et al., 1994). We have good reasons to believe, therefore, that changes in PFC are linked to anti-social and aggressive behavior (Brower and Price, 2001; Sapolsky, 2004; Hirstein et al., 2018). Interestingly, however, Ellenbogen et al. (2005) have reported a case of PFC lesion which resulted in a reverse change, namely a previously anti-social and violent individual turning into a docile and cheerful person (Box 8).

**Box 8. The case of suicide attempt with a crossbow.**

Ellenbogen et al. (2005) describe a case of a suicide attempt with a crossbow. Although the victim, a male in his early 30s, survived, he suffered a severe brain injury, as he shot himself to the head through his lower jaw. The bolt penetrated through the front of the victim’s head, but did not exit through the top of the skull. The result was a prefrontal cortex (PFC) injury, which gave rise to a dramatic personality change. The victim had a record of violent and anti-social behavior. After the injury, however, his behavior changed to the opposite: he became docile, social, and “inappropriately cheerful.” This is in stark contrast to the typically described cases where a lesion to PFC results in aggression and anti-social behavior (Boxes 5–7).

What this evidence suggests, to be precise – and all that it, by itself, suggests – is that there is a connection between the functioning of PFC and social behavior and aggression. Even if there were systematic differences in PFC in people deemed particularly anti-social and aggressive, compared to behaviorally and psychologically normal population, this should not, by itself, lead us to conclude that these people display the anti-social and aggressive behavior due to the changes in PFC, rather than the other way around (cf. Kishiyama et al., 2009). Moreover, the case described by Ellenbogen et al. (2005) indicates that similar types of damages to PFC can actually manifest in completely opposite psychological and behavioral changes (Box 8).

There is, however, one notable issue connecting the cases described above (Boxes 5–7). These are cases where the normal – a particular person’s previous – functioning of the PFC has become disrupted due to an injury (lesion) or a tumor. This is the central reason, it is here suggested, why we can take these sorts of cases to have an impact on our moral and legal reasoning. That is, due to the etiology of these conditions, we do not consider these as cases of “brain rewiring,” and we point to unequivocally biological, physical causes for these conditions. Now, of course, the interesting question is: why do we feel justified in reaching such a conclusion? Several factors are bound to play a role here. For one, lesions and tumors are easy to localize; they are concrete, spatially extended, material entities – something paradigmatically non-mental. They are not fuzzy, and they do not come in degrees, in contrast to the corresponding psychological or behavioral features: nobody has a lesion or tumor “more or less,” but people can be more or less anti-social, or be more or less good at exercising self-control. Considerations related to gradedness in psychological and behavioral features and their neural correlates often affect our moral and legal reasoning by making clear-cut judgments difficult (e.g., Glannon, 2011). In cases of lesions and tumors, however, we can point to precise differences, not only spatially, but also temporally: the observed psychological and behavioral changes are dramatic and sudden, and it is therefore natural for us to tie these changes together with the clearly localized neural changes. These are the reasons, at least some of the main ones, why in cases like these we are prone to point to physical, rather than mental, causes of these conditions.

But we can dig deeper. It is not just that in these cases we feel it is natural to see these conditions as stemming from physical, rather than mental causes, but a radical lack of agential ownership is also associated with the conditions and their causes. That is, it seems clear that one fundamental reason why we find cases like these relevant to our moral and legal reasoning lies in our intuitive feeling that the ultimate source of these conditions must be placed outside the sphere of the influence of the agent in question. But why exactly that is, is not an easy question to answer. In the case of Mr. Whitman, for example, the internal sense of agency seems to have been lost (Box 6). But on the other hand, in the case of Mr. Gage (Box 5), and the case described by Burns and Swerdlow (2003) (Box 7), it is the external sense of agency, or the continuation of personality, that got disrupted. However, in both cases a sudden misalignment of feelings, thoughts, desires, values and actions occurred. This in turn affects the grounds of attributing agency to these subjects, and leads us to place the sources of their conditions outside the scope of their influence.

There is further important element to this way of reasoning. The described cases are cases of lesions and tumors. Lesions and tumors can be considered to be paradigmatic cases of dysfunction and illnesses. That is, in cases of injuries and diseases – such as brain cancer – we are automatically prone to think that something has gone biologically, rather than psychologically, wrong, and we exclude outright the possibility of neural rewiring. These conditions are neurological rather than psychological or psychiatric, and the proper way to intervene on them is physical (surgical, pharmacological) rather than psychological or behavioral. Although this is clearly an important issue affecting our causal and moral reasoning with respect to these cases, one can also envision caveats. The personality change described by Ellenbogen et al. (2005), for example, was due to a self-inflicted brain injury (Box 8). If it were – or when it becomes – possible to change one’s personality traits by direct neural interventions, our attention is bound to shift more from the neural changes to the variety of ways these changes can be induced when taking account of neuroscientific evidence in our moral and legal reasoning [this would parallel the case of “grand schemers” – people who get themselves intoxicated before committing a crime in order to appear less culpable for it (Dimock, 2012)].

There are, therefore, a number of intertwined reasons why in the described cases we find it plausible to let neuroscientific evidence affect our moral and legal reasoning. One could argue, however, that in the case described by Burns and Swerdlow (2003) all the relevant issues come into play in the clearest possible manner (Box 7). Note, also, that this is the only case from the three where the neuroscientific considerations had a real, and significant effect on the proceedings (the case of Mr. Gage has no criminal component to it, and the case of Mr. Whitman never went to trial). In this case, the defendant was in fact acquitted on the basis of the presented neuroscientific evidence (after neuro-surgical interventions had been conducted). Why is this case special? We can point to two reasons. First, it involved a brain tumor, and there are a number of reasons why that has a bearing on our moral and legal reasoning, as just discussed. Second, and more importantly, the tumor could be designated to be the proper difference-making cause of the actions the defendant was accused of: not only did the behavioral patterns considered harmful disappear upon the removal of the tumor, they actually reappeared upon the reappearance of the tumor. This leads us to point unequivocally to the tumor, rather than the defendant, as the source of the actions he was accused of. And, as we hold tumors biological, non-mental entities, paradigmatically dysfunctional in the context of the biology of the person, we place the cause of the actions outside of the scope of the influence of the agent.

## Physicalism, Free Will, and Moral Responsibility

The preceding analysis has been based on the pragmatic assumption of methodological dualism, the idea that it makes sense, in this context, to divide causal explanations into two groups, mental and physical (neural), and, at least sometimes, to point to one of them as the proper cause of behavioral patterns at the expense of the other. This is what we are poised to do when we cite neural changes as the basis of acquittance, as in the case described by Burns and Swerdlow (2003) (Box 7). Note, that in this case [in contrast to the case of Mr. Whitman (Box 6)], the defendant seems to have been motivated in performing the *acti rei*, and the diminished sense of agency was attributed to him on largely on external grounds (although he also stated having attempted to restrain his urges). One could, therefore, argue that the tumor was in fact part of him – his personality – and that it is wrong to see the issue in dualistic terms. That is, one could argue that the appearance of the tumor resulted in physical (neural) changes, which manifested as the personality changes, but that it is wrong to see these two different ways of describing the process as distinct and in competition with each other. However, this is *not* how we, in practice, think. We consider the tumor to be alien to him, creating a biological, and, consequently, a psychological and behavioral dysfunction that calls for correction by physical means (i.e., surgery). So, even though the tumor was part of him – and an essential part of the physical basis of his personality – we take it to be a separate physical factor, and something that deserves, rather than the defendant as a person, to be designated as the cause of the actions deemed harmful.

But there is a deep conceptual problem with such an approach, as has already been stressed: such methodological dualism goes against the metaphysical monism of physicalism. It seems, therefore, that it is not ultimately tenable to hold that we can point to *either* mental *or* physical causes to our actions. Or, more precisely, physicalism seems to make it impossible to hold that there would be unequivocally mental causes to our actions: according to physicalism, all such causes must be physically realized. So, either mental causes – the psychological features we hold causally efficacious – must be reducible to, or identical with, physical causes, or they are not genuine causes at all, and we should altogether refrain from applying causal terminology to the psychological realm. On pain of eliminating our folk psychological practices, on which our moral and legal reasoning rests, we must, therefore, hold all our talk of mental causes to be covert talk of physical causes. But in that case the distinction between the good and the bad ways of applying neuroscientific evidence to moral and legal reasoning, as outlined above, would seem to collapse: in cases where pointing to psychological, social and behavioral factors, rather than to neural factors, as causes of our actions seems to us justified, it will not in fact be so, as we are dealing with physical causal processes through-and-through, and can therefore always point to physical causes of our actions. But if *acti rei* were always performed due to physical causes, and if pointing to such causes function as a basis for exoneration, then all *acti rei* should become exonerable.

This sort of reasoning can be seen to lie behind the more global worries related to the relationship of the neurosciences and jurisprudence (e.g., Greene and Cohen, 2004; Sapolsky, 2004). It is important, however, to make a clear distinction between two different ways of arguing from neuroscientific evidence to conclusions concerning moral and legal responsibility. The global worries are intended to prompt us to entertain doubts about free will and moral responsibility across the board. The argument would start with the assumption – typically a tacit one – that pointing to *any* physical basis for our psyche and behavior should make us cast doubt on moral and legal responsibility, and would then make the further assumption that relying on neuroscientific evidence constitutes such pointing by establishing physicalism. It is clear, however, that this type of argumentation would not make sense as a defense in any individual case, where the goal would be to seek grounds for exonerating the defendant on the basis that she does not have the capacity to exercise control over her actions in the way that is typically taken to be necessary for legal responsibility. In cases of this sort, the evidence only bears on the case insofar as it shows that the defendant is *abnormal* in contrast to typical defendants. But, as has been stressed, merely pointing to neural correlates, by itself, tells us very little about the causal source of the agent’s actions, and on its own provides no grounds for assuming that the factors in question are outside the scope of the agent’s influence. Insofar as global worries are to be taken seriously, then, they would need to be backed up with a much more speculative argument: one aiming to establish that even ordinary neural functioning ought to be regarded as inconsistent with moral and legal responsibility.

Global worries related to the relationship between the neurosciences and jurisprudence are, therefore, unlikely to be of practical importance, at least in individual cases. This explains, partly, why neuroscientific evidence has had much less bearing on actual legal practice than one would maybe have expected. It is worth outlining, however, in a bit more detail, the sort of reasoning that could be seen to give rise to such worries. Note, firstly, that although the discussion here has simply assumed that physicalism holds – that is, it has been assumed that everything is ultimately physical – it is not at all a trivial philosophical project to try to pin down where such an assumption stems from. And indeed, one could argue that physicalism is an empirical thesis, albeit rather holistically and indirectly such, and that the development of the neurosciences, in particular, has played a crucial part in making us reject dualism and persuading us to believe in the monistic metaphysics of physicalism instead (cf. e.g., Papineau, 2001, 2002). It is plausible, therefore, to connect empirical evidence, and the results of the neurosciences in particular, to establishing physicalism. But note that building such a connection is an incremental process, and although some particular results could be seen to bear more significance to it than others – such as connecting electric stimulation to muscle contraction (Galvani, 1791, 1794), or identifying neural cells as the units of the nervous system (Ramón y Cajal, 1888; López-Muñoza et al., 2006), or inventing neuroprosthetic devices (Shenoy et al., 2003; Hatsopoulos et al., 2004; Musallam et al., 2004; Pernu, 2018) – the thesis is not being proved, or disproved, by any single piece of empiria.

However, even if one thinks that there is such a connection between the empirical results of the neurosciences and the metaphysical thesis of physicalism, it is much more contentious to claim that physicalism, by itself, would disprove our ideas of free will and moral responsibility. That might be the case, but it would need to be argued for much more thoroughly and precisely, and it is definitely not a position that would enjoy wide-acceptance in the current discussion – even mental-to-physical reductionism is often motivated by the aim of saving the ideas of mental causation and agency (e.g., Kim, 2005, 2007; Box 3).

Most importantly, however, there is a metaphysical equivocation in here, that the discussion tends to overlook: mental-to-physical reductionism is not psychology-to-neuroscience reductionism. That is, even if we were to subscribe to a thoroughly physicalist metaphysics, we would not be committed to the idea that by inspecting the brains – or even the whole bodies – of people, we can in an any meaningful way, let alone perfectly, read their psyche. What we are facing here is the very same problem we have been facing all along: our brains, and our bodies, are built to react to environmental cues. So, whether a bodily state – a physical state – represents something meaningful, is not something that hinges on that bodily state alone. The context matters; the environment in which the body resides – and has resided – needs to be taken into account. Mental dysfunctions, in particular, are highly sensitive to various environmental factors. The problem is not just that the mind can be multiply realized by various different bodily states – that you cannot read the bodily state from the mental state – but that in fact the opposite holds too: that the same bodily state can realize various different mental states – due, simply, to neural plasticity and reuse – and you cannot, therefore, identify the mental state by simply nailing down the exact bodily state that happens to realize it [as illustrated by the varied psychological and behavioral changes resulting from PFC lesions, aggressive and anti-social cases (Boxes 5–7) in contrast to docile and social cases (Box 8)]. The surroundings of the body – and not just the current stimuli it receives, but all the environmental cues that it has historically been exposed to – play a crucial role in shaping the body. Neither the bodies, nor the brains as their proper parts, can, therefore, play the role of a proper, complete physical realizers of mental states.

Consider, to make this argumentation more concrete, the famous case of *Illinois v Leopold and Loeb* (Box 9). The case is known – apart from the morbidity of the crime – precisely for the issue of global worries related to free will being presented to, and having an effect on, the court. It is notable, however, that even though the case is also important for it being one of the first examples of biological and neuroscientific evidence being presented to the court as a basis of the culpability assessment of the defendants (Weiss, 2011; Wilson, 2015), this did not, once again, have an effect on the final decision of the court. What did play a part in the ruling, instead, were more general environmental and social considerations, related to the age of the defendants in particular. In this way the case is actually quite strongly analogous to the more recent case of *Roper v Simmons* (Box 4).

**Box 9. The people of the State of Illinois v Nathan F. Leopold Jr. and Richard Loeb 33623/33624.**

**FIGURE 5:**
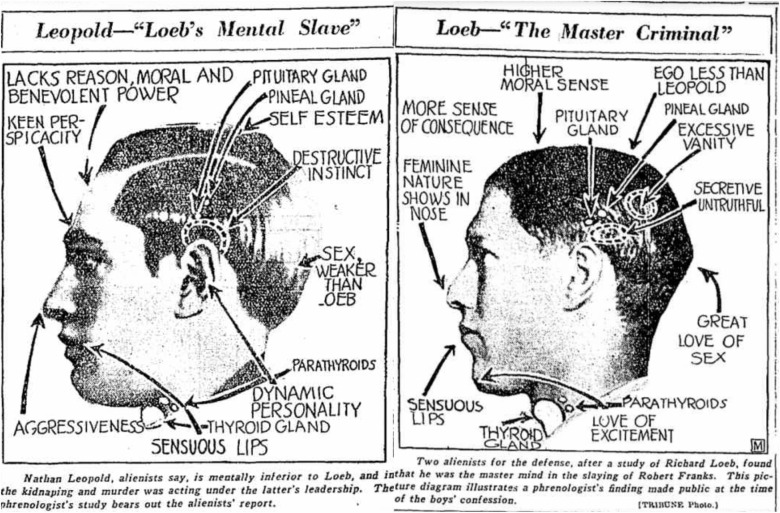
Psychiatrists’ (“alienists”) phrenological assessments of Mr. Nathan Leopold and Mr. Richard Loeb as they appeared in The Chicago Tribune in the summer of 1924.

One of the most infamous cases in criminal history (e.g., Higdon, 1975; Theodore, 2007) occurred in Chicago in May 1924, when 19-year-old Nathan Leopold (1904–1971) and 18-year-old Richard Loeb (1905–1936) conspired in the kidnapping and murder of Robert “Bobby” Franks (1909–1924), a 14-year-old neighbor and second cousin of Loeb. Leopold and Loeb were students at The University of Chicago, both wealthy and high academic achievers, with Leopold often described as a child prodigy, and with Loeb skipping ahead many years in school and becoming the youngest graduate of the University of Michigan at the age of seventeen. They spent several months planning the kidnapping and murder of their victim, and they were determined to commit “the perfect crime,” simply for the thrill of it. They were inspired by the works of Friedrich Nietzsche (1844–1900), with Leopold supposing that their superior intelligence meant that they were “Übermenschen” and above the social and moral conventions that bound average, unexceptional people. Despite their efforts to make sure they would not get caught, the police quickly found leads that pointed to the boys’ guilt, and they soon confessed to the crime. The trial at the Chicago’s Cook County Courthouse courted sensational media coverage during the summer of 1924. The defendants hired a renowned defense attorney, Clarence Darrow (1857–1938), who was an outspoken opponent of capital punishment. He entered a guilty plea, but proceeded to persuade the judge to avoid sentencing the defendants to death. The court proceedings of the case are interesting in two respects. First, this is one of the first criminal cases where psychological and neuroscientific evidence was presented in a trial (Weiss, 2011) – albeit some of it in a now debunked form of phrenology (Figure 5). Second, the concluding speech of the defense, presented by Darrow, is famous for its sentiment, rhetoric, and appeals to global worries about free will. The closing argument, which lasted for twelve hours, built such an emotionally strong case for the defendants that it left the judge himself in tears. Argumentatively, the speech was based on Darrow’s conviction that none of us are really the sources of our choices, but they, and all the actions we base on our choices, are rather fully determined by psychological, physical, and environmental factors outside the scope of our influence (Darrow, 1922). In the trial, Darrow therefore pleaded that the boys ought to be spared on grounds that focused primarily on societal issues, making relatively little reference to the unique circumstances of the defendants in committing the murder [it is notable, however, that according to some of the expert witnesses of the defense the defendants were emotionally impaired, and Darrow would later argue that proper emotional functioning is necessary for making well-founded choices (Darrow, 1932) – echoing some of the developments that have been taking place in the discussion in the last 20 years or so]. E.g., the speech drew on the claim that, in the aftermath of the First World War, society had increasingly glorified war, sending a message to young people that life is cheap and killing is trivial. The core of Darrow’s argument is neatly summarized in the following passage from the speech:

“Why did they kill little Bobby Franks? Not for money, not for spite; not for hate. They killed him as they might kill a spider or a fly, for the experience. They killed him because they were made that way. Because somewhere in the infinite processes that go to the making up of the boy or the man, something slipped, and those unfortunate lads sit here hated, despised, outcasts, with the community shouting for their blood” (Darrow, 1924, p. 22).

The speech was successful: instead of death by hanging, Leopold and Loeb were sentenced for a life in prison plus 99 years.

Loeb was killed in prison by a fellow inmate in 1936. Leopold was eventually released in 1958, and he completed a master’s degree at the University of Puerto Rico, after which he worked in various teaching posts and research projects – even publishing a book on ornithology (Leopold, 1963). He died in Puerto Rico from natural causes in 1971.

None of this should make one conclude that physicalism does not hold. It should only prompt one to reject the idea that mind-body reductionism holds. To have a complete, firm grip on the mind, being in possession of a complete physical description of the body is not enough. You also need to be in possession of the complete description of the body’s surroundings, and the history of the interactions of the two. The mind – its content – in other words, is, in physical terms, not only dependent on the nervous system that happens to realize it, but also on the environment to which that nervous system has been adapted. All this can, in principle, be described in physical terms, as both the body and its surroundings are, in the final analysis, physical entities. In practice, however, the interactions are too intricate, and the system as a whole is too complex, for us to be able to make sense of it in purely physical terms.

The mental might, therefore, be reducible to the physical, but it won’t be reducible to mere bodily states. That is why the global worries related to the relationship between the neurosciences and our moral and legal reasoning are largely misguided. Even if physicalism holds – as has been assumed here – the neurosciences, by themselves, will not unravel all the physical bases relevant to our psyche and behavior. To accomplish that, the neuroscientific evidence would need to be supplemented by a plethora of other physical information; rather paradoxically, the more physically detailed information of people and their various interactions we gather, the less relevant purely neuroscientific evidence will become. It is clear, therefore, that *neuro*law will never pose a threat to our folk psychological ways of doing moral and legal reasoning. But *physical* law still might. It is reasonable to assume, however, that we will never get there.

## Conclusion

The mind is dependent, in a crucial way, on its biological basis, the nervous system in particular. Information about this basis should, therefore, have a straightforward impact on our moral and legal reasoning, and, ultimately, on practical jurisprudence. However, despite advances of the neurosciences, neuroscientific evidence has not played a significant role in recent legal cases. Why is that?

Fundamentally, it has here been argued, this is due to the discussion conflating a number of separate issues. As we already know that minds are dependent on brains, finding neural correlates of our psyche and behavior should not come as a surprise to us. Yet, the findings are often portrayed as such. This dualistic – fallacious – sentiment is present also in the discussion on the impact of the neurosciences on jurisprudence. Although we can often point to clear neural changes as being associated with the sort of a behavior, *actus reus*, that is under scrutiny in court proceedings, it is wrong to think that we should conclude that these neural changes are causally responsible for the behavior in question. All behavior has a neural basis, not only the sort that we find morally or legally concerning.

We need, therefore, some independent, and ultimately psychologically and socially based, grounds for thinking that a particular neural change or feature is of such a sort that it should be designated as a cause of some behavior. When deeming a biological basis of decisions and actions dysfunctional, we need to employ psychological and social considerations: it is on the basis of our prior, and often very basic and intuitive psychological and social knowledge that we come to suspect that there is something biologically peculiar in some people, and not the other way around. Only in some rare, very clear cases of externally caused brain lesions are we prone to designate some unequivocally neural changes as causes of *acti rei*, and to exonerate defendants on the basis such evidence.

Why, then, does neuroscientific evidence of various sorts continue to be presented in court proceedings? Precisely because we are convinced that our psyche and behavior are ultimately neurally based. But even if it were taken for granted, as it here has been, that physicalism holds, and that all our mental states are necessarily dependent on their neural basis, it would be wrong to think it is only neural evidence that we need to rely on to give a complete account of our psyche and behavior. To do that – to completely explain in physical terms some particular mental or behavior features – a more encompassing physical account of the person and her history needs to be given.

We are yet to fully comprehend our nature as thoroughly physical beings in a perfectly physical context. Maybe someday the sciences will paint such a complete picture of us and the surrounding world for us, and maybe that will lead us to abandon the very idea of free will, and the notions of moral and legal responsibility that seem to require such an idea. Whether that is what will indeed happen, is not, however, something that we are in a position to predict. But whatever the verdict will be, it is clear that it is not something that will be reached in some legal process in a particular court of law. It is something that will be reached in the gradual process of all the sciences providing us with a unified understanding of us as conscious, intentional and moral beings.

## Author Contributions

Both authors listed have made a substantial, direct and intellectual contribution to the work, and approved it for publication.

## Conflict of Interest

The authors declare that the research was conducted in the absence of any commercial or financial relationships that could be construed as a potential conflict of interest.

## Footnotes

1 To be clear, taking these notions to be connected in this hierarchical way does not entail that one is committed to some realistic or naturalistic way of interpreting the notions of moral and legal responsibility (cf. Moore, 1984). The thesis is merely that these notions are this way connected, whatever their ontological status. In particular, this does not commit one to rejecting legal positivism (and embracing the natural law view). One could hold that law (and morality – and causal explanation for that matter) are wholly socially dependent entities, but still maintain that they are connected in the manner outlined here. Note also that *any* view on law and morality must distinguish between them, and give an account of their connections. Indeed, it is one deeply entrenched misconception that legal positivism would be committed to the complete severing of the connection between law and morality (cf. Gardner, 2001).
